# The Value and Significance of Metagenomics of Marine Environments

**DOI:** 10.1016/j.gpb.2015.10.002

**Published:** 2015-11-27

**Authors:** Fangqing Zhao, Vladimir B. Bajic

**Affiliations:** 1 Computational Genomics Lab, Beijing Institutes of Life Science, Chinese Academy of Sciences, Beijing 100101, China; 2 Computational Bioscience Research Center (CBRC), Computer, Electrical and Mathematical Sciences and Engineering Division (CEMSE), King Abdullah University of Science and Technology (KAUST), Thuwal 23955-6900, Saudi Arabia

Microbes have played a fundamental role in the natural history of our planet, and have done so for billions of years. They have adapted to Earth’s many environments from the mild to the very extreme. Studying their diversity and their way of life is critical for understanding their full impact on the global ecology. Although the field of metagenomics is still young, it has unravelled a wider microbial diversity that had otherwise been completely overlooked by the traditional methods of microbiology. There are three major metagenomic themes: (a) marker metagenomics that surveys microbial community structure by targeting the highly-conserved 16S rRNA gene, (b) functional metagenomics that takes the total environmental DNA, from which it infers the metabolic potential of the microbial community, and (c) identification of novel enzymes.

Functional metagenomics has added to our understanding of microbial ecology and their role in the global geochemical cycles. Some of the most important contributions from this field include the discovery that proteorhodopsin is widespread in marine environments [1], which explains how bacteria are able to thrive in oligotrophic environments. Another exciting discovery is the recovery of ammonium monooxygenase from an archaeal genomic fragment where until then it was believed to be exclusive to bacteria [1], [2]. Metagenomics has also led to several novel insights into the various geochemical cycles including phosphorus, sulfur, carbon, and nitrogen.

Comparing genomic information of microbes from different environments provides evidence for their niche adaptations. The landmark metagenomic sampling that was carried out in the Sargasso Sea revealed high microbial diversity [1], while the microbial community that was reported in another landmark metagenomic study based on samples from an acid mine drainage revealed a far less complex community [3]. Other metagenomic studies that compared the metabolic potential of microbial communities from differing environments, *e.g.*, agricultural soil, sea surface, and deep-sea whale carcasses, have shown noticeable differences in the enrichment of various genes that supports microbial lifestyle in their niche [4], [5].

The field of metagenomics has allowed us to tap into a vast microbial diversity that has, for long, eluded the traditional microbiology methods. Beyond simply counting new species, metagenomics has also helped establish the link between the gene pool available to a microbial community and the environmental parameters that surround them. The explosion in metagenomics data, especially those from extreme environments, has provided a fresh source for novel biocatalysts that may be of value to the biotechnological sector [6].

The journal *Genomics, Proteomics & Bioinformatics* (GPB) has compiled a Special Issue on Metagenomics of Marine Environments. We have selected seven papers for inclusion in this special issue. The accepted contributions cover various aspects of relevance, from methods, tools, and resources to specific studies of different metagenomic samples.

Zhang and Ning [7] describe the opportunities and challenges related to the mass generation of metagenomics data (>70 TB) from the *Tara* Oceans Project. To date, 30,000 samples from 200 base stations containing millions of organisms have been collected. As a result, novel sequences from viruses, prokaryotes, and pico-eukaryotes have been discovered as reported in [8]. Furthermore, new knowledge on oceanic eco-systems was acquired, for instance, the key role that temperature and oxygen levels play in determining microbial community composition compared to others such as salinity [9]. Local and global patterns were found to have a similar effect on plankton interaction [10]. Additionally, a core-gene set for the upper-ocean viral community has been constructed [11]. The authors describe some interesting opportunities available from this project such as identifying proteins that have unusual functions and assessing the difference in sequence composition between orthologous proteins from the land and the deep ocean. In addition, the large amount of data could improve existing reference assemblies and annotation of pathways. An example of this is the potential analysis of photosynthetic pathways between different samples (*e.g.*, samples from upper and deeper levels of the ocean). Major challenges facing the *Tara* Oceans Project are poor data and information management, which is easily solved with proper database and storage architecture. The other issue mentioned by the authors is the lack of optimized statistical models.

Rhoads and Au [12] describe the applications, advantages, disadvantages, and future of PacBio sequencing vis-à-vis second-generation sequencing (SGS). Due to its advantages, PacBio sequencing has a potentially great role in metagenomics. SGS is frequently unable to close gaps in draft genomes, especially those with high repeat content due to the short read length. On the other hand, PacBio sequencing is increasingly being used to close gaps in previously-unfinished reference assemblies and in novel ones [13]. PacBio is used to characterize structural variations [14] such as copy number variations (CNVs) and long insertion-deletions (INDELs). It facilitates reliable discoveries of novel genes [15] and isoforms [16], and is assisting in the detection of base modifications and other aspects of epigenetics. PacBio produces longer read lengths and faster run-times compared to SGS but at the expense of throughput and accuracy. *De novo* assembly of genomes using PacBio alone is costly and therefore most use a hybrid approach that combines two or more sequencing platforms. Examples are higher detection rate of structural variations [17] and identification of gene isoforms [16]. The authors also briefly compare PacBio sequencing with other third-generation sequencing (TGS) such as Oxford Nanopore [18], showing a poor accuracy rate of Oxford Nanopore albeit producing longer average reads. Lastly, authors suggest that the new Sequential System by PacBio could potentially reduce the cost and increase throughput over the current RS II system.

The article by Alma’abadi et al. [19] reviewed marine metagenomes as a potential source for novel industrially-useful enzymes. With the advance of the next-generation sequencing (NGS) technologies and associated studies of metagenomes, we have now realized the extent of the unknown biocatalysts. Indeed, the majority of microbes identified in metagenomic studies cannot, so far, be cultured. This apparent lack of capability by the traditional laboratory techniques is somewhat compensated for by the wealth of data provided by the metagenomics techniques. Researchers can tap into the vast swathes of metagenomic data not only to answer questions such as “who is there” and “what are they doing”, but also to discover naturally-evolved biocatalysts that can drive environmentally friendlier industries. Alma’abadi et al. use lipases as an example to drive home the role of metagenomics in the discovery of novel enzymes. The authors also delve into the experimental and computational difficulties that currently limit the potential of such techniques.

The contribution by Dudhagara et al. [20] is a mini review of 12 most cited online resources for metagenomics studies. The significant advances made in the field of NGS combined with the ever decreasing per base cost have exploded in an ocean of data. New challenges have sprung up for the bioinformatics side of the equation. Not only the current bioinformatics algorithms have to adapt to the new specifications (short reads, higher error rates, and technology-dependent), but the availability of the technology to smaller laboratories, which cannot afford a full-time bioinformatician and do not own the hardware necessary to analyze such data, strongly urges and promotes the development of cloud-based resources. Such resources must capture the complexity of the underlying data yet be intuitive and easy to understand to the non-informatics-savvy scientist. This review by Dudhagara et al. should serve as a handy resource to newcomers to the field of metagenomics. It describes 12 online resources and ranks them according to their citations, showing that the most widely used tool is MG-RAST, followed by IMG-M and MetaRep.

Antunes et al. [21] looked into the viral communities present in the deep-sea brines of the Red Sea. These unusual extreme environments have been the targets of several recent studies aiming to elucidate their microbiology (*e.g.*, [22], [23]), but none of these have looked into their viral communities. Antunes et al. explored four metagenomic datasets from the brine-seawater interface as a first step to close this knowledge gap. The authors report on very diverse and stratified viral communities, which are distinct from sample to sample. Despite being generally dominated by Caudovirales, this study detected high numbers of unclassified and environmental viruses (particularly pronounced for the Atlantis II brine pool), and unexpected hits for Phycodnaviridae and Iridoviridae. These findings provide important first insights into the unexplored viral communities present in deep-sea brines of the Red Sea and constitute the first step for ongoing and future sampling efforts and studies.

Simões et al. [24] report on the fungal communities present in gray mangroves of the Red Sea. The scarcity of data available from these locations [25], or even from general rhizosphere-associated fungal communities, makes this a particularly pertinent study. Simões et al. uncovered that Ascomycota dominated, yet Basidiomycota were present in higher numbers than usually reported. This metagenomics-based study revealed that overall, fungal communities of the gray mangroves of the Red Sea are significantly richer than previously assumed, representing unique, under-explored sources of fungi with potential relevance in the fields of biotechnology, food industry, and health research.

In the Application Note section, Zuo and Hao [26] describe the improvements made to the whole genome-based phylogenetic tree builder, CVTree version 3.0. Given a whole genome, it can predict the phylogeny of the genome without the hassle of identifying orthologous proteins. There are many improvements made to the latest release of CVTree. First, unlike previous releases, the peptide length, K, is not required to be pre-defined. Instead, a range of K is calculated and the best is chosen. Next, the algorithm in release 3.0 is able to utilize the power of parallel computing as the web-server now resides on a 64-core server. CVTree’s new interactive display enables the study of both taxonomy and phylogeny, because of the ability to collapse and expand trees interactively. Another new feature is the ability to report the number of genomes from all taxa in all ranks (domain to species). The built-in database of CVTree has been further improved by the addition of many genomic databases especially for prokaryotes. Unlike the previous releases, which only use NCBI datasets, release 3.0 integrates datasets from the European Nucleotide Archive (ENA), International Microbial Genome (IMG), Broad Institute, J. Craig Venter Institute, Pathosystem Resource Integration Center Microbial Dark Matter Project, and many others. The authors performed a retrospective classification of CVTree3 on recent prokaryotic classification/re-classification and found no contradictions.

In conclusion, we believe that this Special Issue makes useful contribution to the field of Metagenomics of Marine Environments and that it will serve as a valuable resource, containing easy to follow material useful to researchers in this field.

## Competing interests

The authors declared that there are no competing interests.
